# The Brief Case: Incidental finding of a liver fluke following resection of hepatocellular carcinoma

**DOI:** 10.1128/jcm.01302-24

**Published:** 2025-01-31

**Authors:** Matthew Chung Yi Koh, Kevin Mong Sheng Sim, Blaine A. Mathison, Richard S. Bradbury, Jinghao Nicholas Ngiam, Nicholas Jian Hao Chan, Jolene Ee Ling Oon, Aileen Wee, Gabriel Zherong Yan

**Affiliations:** 1 Division of Infectious Diseases, Department of Medicine, National University Health System 150744 , , Singapore; 2 Department of Pathology, National University Hospital 59053 , , Singapore; 3 Department of Pathology, The University of Utah 161530 , Salt Lake City, Utah, USA; 4 School of Public Health and Tropical Medicine, James Cook University 8001 , Townsville, Queensland, Australia; 5 Yong Loo Lin School of Medicine, National University of Singapore 37580 , , Singapore; 6 Division of Microbiology, Department of Laboratory Medicine, National University Hospital 59053 , , Singapore; Mayo Clinic Minnesota, Rochester, Minnesota, USA

**Keywords:** liver fluke, *Clonorchis sinensis*, hepatocellular carcinoma, incidental finding, hepatitis B virus, non-endemic

## CASE

A 76-year-old male with chronic hepatitis B virus (HBV) infection was on follow-up with the gastroenterology clinic for abnormal findings on liver imaging. His HBV infection had been diagnosed 8 years prior; he was currently on entecavir, and his HBV viral load had been suppressed for years. Six months prior, he was found to have a 1.7-cm lesion in the right lobe of the liver on routine surveillance ultrasound imaging. He was asymptomatic at the time.

This lesion was followed up with magnetic resonance imaging, which showed a 4-cm lesion in segment VII of the liver. This demonstrated diffuse arterial enhancement with washout, concerning for hepatocellular carcinoma (HCC). He remained asymptomatic. Full blood count examination showed a normal total white blood cell count with no eosinophilia. Liver function testing was also unremarkable. Alpha-fetoprotein was not elevated.

**Fig 1:**
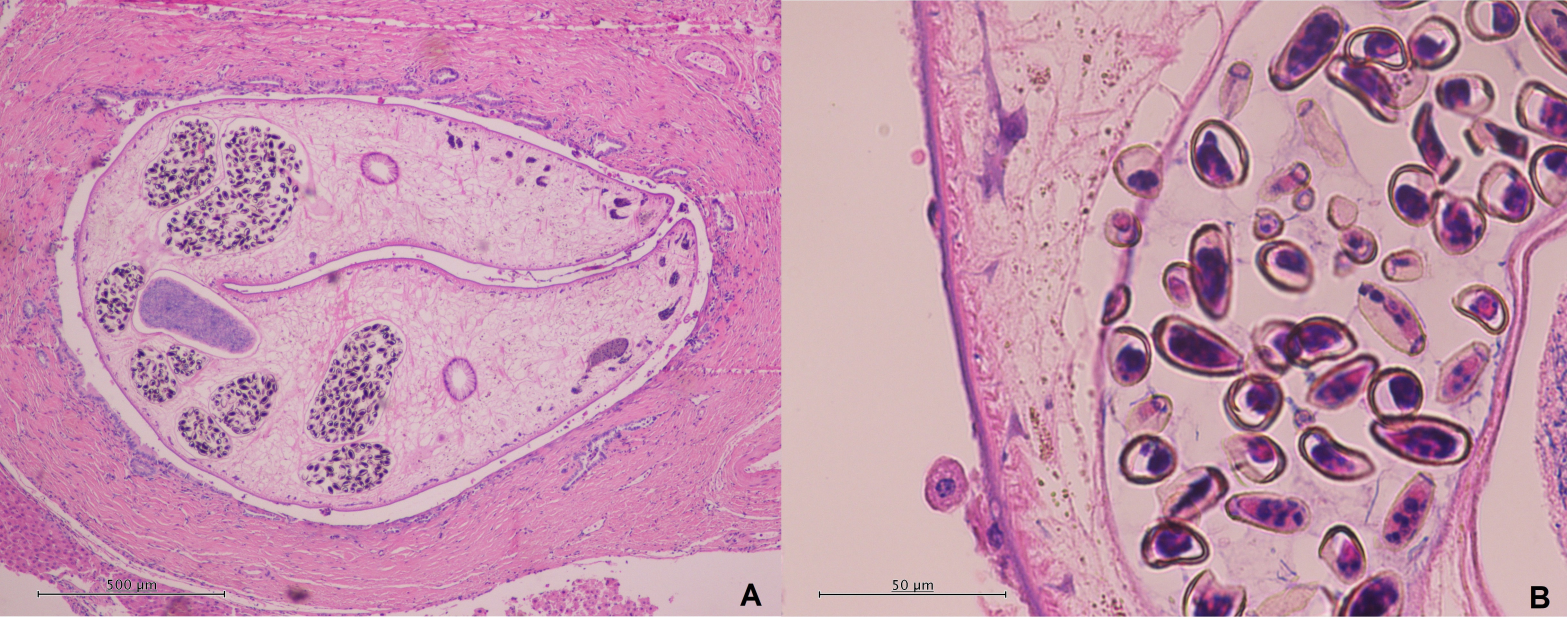
(A) Histopathology of liver segmental resection demonstrating gravid fluke (width approximately 3 mm) within a hilar bile duct. Innumerable eggs found within its uterus. Adenomatous hyperplasia of the duct was found with no dysplasia or malignancy. (Hematoxylin-eosin stain; original magnification ×10.) (B) Eggs, with largest measuring approximately 24 × 11μm, within the uterus of the liver fluke. (Hematoxylin-eosin stain; original magnification ×40.)

Therapeutic options were discussed with the patient, and he elected for definitive surgical resection of the lesion. He underwent an open right posterior sectionectomy of the liver. Histopathological examination confirmed the presence of a well- to moderately differentiated HCC. Incidentally, a gravid fluke with a width of approximately 3 mm was also found within a hilar bile duct (Fig. 1A). Its uterus contained innumerable eggs, the largest measuring approximately 24 × 11 µm (Fig. 1B). Adenomatous hyperplasia of the associated bile duct was demonstrated, but no evidence of dysplasia or malignancy besides the aforementioned HCC was found. Additionally, eggs were also found scattered within the liver parenchyma, separate from the adult liver fluke.

The patient was a Singaporean resident. Five years prior to his current presentation, he had traveled to Wanchang in rural Hainan province, China, and had consumed local food while there. Seven years prior, he had traveled and stayed within the urban regions of Beijing, Shanghai, and Shenzhen in China. More recent travel included visiting urban areas in Sydney (Australia), Bangkok (Thailand), and Kuala Lumpur (Malaysia) in the year prior to presentation. Dietary-wise, he reported consuming raw salads regularly. He could not recall previously consuming raw or undercooked freshwater fish or snails. He did not report any history of chronic abdominal pain or diarrhea, fever, or recurrent biliary infections.

Based on the morphological features on histopathology of the fluke, including the dimensions of the adult fluke and its eggs, as well as the epidemiological history of previous travel to China, a presumptive diagnosis of asymptomatic infection with *Clonorchis sinensis* was made. Given its association with cholangiocarcinoma, even with asymptomatic clonorchiasis, the patient was offered further evaluation and anthelmintic therapy with oral praziquantel 25 mg/kg three times a day for 2 days. His wife, who shared the same risk factors as him travel- and dietary-wise, was managed similarly. Microscopic examination of three stool samples from both patients was performed, but neither the eggs nor the adult worms were found.

## DISCUSSION

Flukes are multicellular parasites of the phylum Platyhelminthes, class Trematoda, whose adult members display tissue tropism in infections of humans. Common flukes that cause parasitism of the human liver and biliary tracts include the causative agent of human clonorchiasis, *Clonorchis sinensis*, flukes of the genera *Opisthorchis*, *Opisthorchis viverrini*, and *Opisthorchis felineus*, as well as *Fasciola hepatica* and *Fasciola gigantica*, flukes that cause human fascioliasis (1). Table 1 summarises the characteristics of these common liver flukes (1, 2). Opisthorchid flukes, which include *Clonorchis* and *Opisthorchis*, have three main hosts in their life cycle, with freshwater snails as the first intermediate hosts and freshwater fish, most commonly of the Cyprinidae family, as their second intermediate hosts. Humans become infected and act as the definitive hosts when they consume such raw or undercooked freshwater fish, which contain the metacercaria of the flukes (3, 4). The metacercaria then excyst in the duodenum, with juvenile flukes migrating to intrahepatic biliary ducts where they develop into adults (1, 3, 4).

**TABLE 1:** Comparative characteristics of common liver flukes

Liver fluke	First intermediate host (genus)	Second intermediate host	Mode of transmission	Geographic distribution	Adult fluke size (mm)	Egg size (µm)
*Clonorchis sinensis*	Freshwater snails (*Bithynia*, *Parafossarulus*)	Freshwater fish	Ingestion of raw or undercooked fish	China, South Korea, Taiwan, Northern Vietnam, Eastern Russia	Length: 8.0–25.0 Width: 1.5–5.0	Length: 26–35 Width: 12–19
*Opisthorchis viverrini*	Freshwater snails (*Bithynia*)	Freshwater fish	Ingestion of raw or undercooked fish	Southeast Asia (Thailand, Laos, Cambodia, Central and South Vietnam)	Length: 5.5–9.5 Width: 0.7–1.7	Length: 22–32 Width: 11–22
*Opisthorchis felineus*	Freshwater snails (*Bithynia*)	Freshwater fish	Ingestion of raw or undercooked fish	Eastern Europe, Russia	Length: 8.0–18.0 Width: 1.2–2.5	Length: 21–36 Width: 11–17
*Fasciola hepatica*	Freshwater snails (*Lymnaea*, *Galba*)	Watercress and other aquatic plants	Ingestion of contaminated aquatic plants	Worldwide (common in Eurasia, parts of South America, Africa)	Length: 13.0–31.0 Width: 5.0–15.0	Length: 130–150 Width: 63–90
*Fasciola gigantica*	Freshwater snails (*Radix*, *Lymnaea*)	Watercress and other aquatic plants	Ingestion of contaminated aquatic plants	Tropical regions of Africa, Asia	Length: 25.0–75.0 Width: 5.0–12.0	Length: 150–190 Width: 70–90

In this case, the liver fluke was found incidentally on histopathology while the host was asymptomatic, a feature not uncommonly seen with human clonorchiasis. Hosts with early or light infections with *C. sinensis*, in which there is a low fluke burden and eggs per gram of stool, are rarely symptomatic. With heavier parasitic loads, patients experience symptoms such as abdominal pain and diarrhea or suffer complications of the biliary tract such as cholelithiasis or cholangitis (4, 5). Of significant concern is also that *C*. sinensis is a World Health Organization (WHO) recognized Group 1 Carcinogen. Chronic infections are associated with the development of cholangiocarcinoma, with pooled odds ratio of approximately 4.5 reported in meta-analysis (6).

*C. sinensis* is predominantly found in East Asia, with 85% of cases reported from China, and smaller proportions found in the neighboring regions of South Korea, northern Vietnam, and far eastern regions of Russia (6, 7). In contrast, *O. viverrini* is found in Southeast Asia, while *O. felineus* can be found in Eastern Europe and Russia. In non-endemic regions, infections are usually found in immigrants originally from endemic regions or in travelers to endemic countries who have eaten undercooked freshwater fish (4, 6). In this case, the previous history of travel to China was vital in establishing a provisional diagnosis of the fluke, although a clear foodborne exposure was not identified. *C. sinensis* infections can have a long latency, with cases reported in immigrants from endemic regions who migrated to non-endemic countries up to 50 years previously (8, 9). *C. sinensis* is not known to be endemic in Singapore, with only a few sporadic cases reported in the literature. Interestingly, an early case report described a Singaporean male, who died of heart failure, who had *C. sinensis* eggs found in stool and adult flukes demonstrated in the liver posthumously, in the absence of previous travel to endemic regions. An attempt was made to evaluate the local freshwater carp this gentleman ate for *C. sinensis* but this was unyielding (10).

Co-infections of *C. sinensis* with HBV have been reported and can be attributed to their geographical overlap in endemic regions of East Asia (11). Diagnosis of either condition is made more challenging in co-infections given the overlap in their hepatobiliary manifestations. Treatment can also be more complicated, with some authors reporting poorer responses to HBV antiviral therapies in co-infected patients (12, 13). However, this has yet to be consistently found, and it remains inconclusive if *C. sinensis* and HBV co-infections are synergistic in their capacity for hepatitis or development of hepatobiliary malignancies such as HCC or cholangiocarcinoma (11, 13–15). Infections with *C. sinensis* alone are not known to be associated with HCC. In our patient, it was perhaps fortuitous that the patient underwent surgical resection of his HCC as this allowed for diagnosis of his otherwise asymptomatic fluke infection. Although the presence of only one fluke on histopathology and lack of further eggs found in stool specimens suggested a low burden of infection, this did not preclude a potentially more widespread subclinical infection, especially given the presence of eggs scattered across the liver parenchyma, and not just within the adult liver fluke in the bile duct. Anthelmintic treatment was prescribed as a precaution for a theoretical risk of future hepatobiliary complications as well as the development of a potential second malignancy in the form of cholangiocarcinoma.

Morphological features of the fluke on histopathology also aided with the provisional diagnosis in this case. The size and aspinous tegument of the fluke in our case made flukes of the genera *Fasciola* unlikely. Adults of *C. sinensis* tend to be about 8–25 mm long and 1.5–5.0 mm wide with eggs about 26–35 μm long and 12–19 μm wide (2). Figure 1A demonstrates the fluke in cross section with a width of approximately 3.0 mm with Fig. 1B showing eggs within its uterus measuring up to approximately 24 × 11 µm. As compared to flukes of the genera *Opisthorchis*, *C. sinensis* often demonstrates testes which are much more branched; however, this was not visible in the cross section we were able to obtain on histopathology. Despite the above morphological features identified in the fluke on histopathology, a clear diagnosis remained challenging given potential overlap with *Opisthorchis* spp., and the current lack of accredited serological testing. The history of epidemiological exposures was therefore vital in helping establish the diagnosis, given the different endemic geographic regions of the various liver flukes.

A variety of anthelmintic agents and dosing regimens have been studied for *C. sinensis* infections, but we elected to give our patient and his partner the WHO-recommended regimen of oral praziquantel 25 mg/kg three times a day for 2 days. In meta-analysis, this regimen has shown a high predicted cure rate of 98.5%, with a 95% CI of 85.4%–99.0% (16). Alternative agents include albendazole and tribendimidine. Treatment of household contacts as in this case can be considered; however, the basis of this is not human-to-human transmission but rather shared epidemiological exposures. Vinh et al. performed a social network analysis study in four villages in North Vietnam which found that risk for *C. sinensis* infection in household clusters was increased by residents eating more raw fish due to participation in social networks which shared fish caught from nearby rivers (17). In our case, although a definitive diagnosis of clonorchiasis was not established in the patient’s wife given the lack of eggs found in her stool samples, anthelmintic therapy was offered given the low potential risk of treatment as compared to the potentially devastating future complication of cholangiocarcinoma.

This case highlights the incidental finding of a gravid fluke, likely *C. sinensis*, during surgical resection of HCC in a gentleman with chronic HBV infection. Morphological features on histopathology, together with a history of epidemiological exposures, proved important in establishing a provisional diagnosis of the fluke. Cholangiocarcinoma is an important potential complication of *C. sinensis* and should be part of the consideration when deciding on offering anthelmintic therapy for asymptomatic patients.

## SELF-ASSESSMENT QUESTIONS

1. In which of the following areas is clonorchiasis most common?
  - East Asia
  - South America
  - Central Africa
  - North America
2. Which of the following cancers is a potential complication of *Clonorchis sinensis* infection?
  - Hepatocellular carcinoma
  - Cholangiocarcinoma
  - Gastric adenocarcinoma
  - Urothelial carcinoma of the bladder
3. What is the recommended treatment option for clonorchiasis?
  - Ivermectin
  - Triclabendazole
  - Diethylcarbamazine
  - Praziquantel

## ANSWERS TO SELF-ASSESSMENT QUESTIONS

1. In which of the following areas is clonorchiasis most common?
  - East Asia
  - South America
  - Central Africa
  - North America

Answer: a. *Clonorchis sinensis* infections are predominantly reported from East Asia, with the majority of cases from China. Smaller proportions of cases have been reported from regions such as South Korea, northern provinces of Vietnam, and far eastern regions of Russia. *C sinensis* is not known to be endemic to Singapore. Reported cases in non-endemic areas are usually in travelers to and from endemic countries with possible previous ingestion of raw or undercooked freshwater fish.

- Which of the following cancers is a potential complication of *Clonorchis sinensis* infection?
  - Hepatocellular carcinoma
  - Cholangiocarcinoma
  - Gastric adenocarcinoma
  - Urothelial carcinoma of the bladder

Answer: b. *C. sinensis*, together with flukes of the genera *Opisthorchis*, has been associated with the development of cholangiocarcinoma. Hepatocellular carcinoma is not known to be a direct consequence of *C. sinensis* infections but rather can develop following chronic infections with hepatitis B or C viruses. Gastric adenocarcinomas are associated with infection with the bacteria *Helicobacter pylori*. Chronic infections with the fluke *Schistosoma haematobium* can cause urothelial carcinomas of the bladder.

- What is the recommended treatment option for clonorchiasis?
  - Ivermectin
  - Triclabendazole
  - Diethylcarbamazine
  - Praziquantel

Answer: d. The WHO-recommended regimen for treatment of clonorchiasis is oral praziquantel 25 mg/kg three times a day for 2 days. This regimen achieves predicted cure rates of 98.5%, with a 95% CI of 85.4%–99%. Other potential treatment options for clonorchiasis include albendazole or tribendimidine. The other listed options are ineffective for clonorchiasis. Ivermectin is used in the treatment of other helminths including strongyloidiasis and ascariasis. Triclabendazole is the anthelmintic of choice for fascioliasis and can also be used as an alternative option for paragonimiasis. Diethylcarbamazine is effective for lymphatic filariasis, loiasis, and visceral larva migrans.

**TAKE-HOME POINTS**

- *Clonorchis sinensis* should be suspected in travelers to and immigrants from endemic countries such as China, South Korea, or Vietnam. Clonorchiasis can have a long latency, and even remote histories of previous travel to such regions many years prior, especially with consumption of raw freshwater fish, should be explored.
- Early or light infections with *Clonorchis sinensis*, in which there is low fluke burden and minimal to no eggs per gram of stool, can often be asymptomatic.
- Cholangiocarcinoma is an important and potentially devastating complication of *Clonorchis sinensis* infections, which should be taken into consideration when deciding on offering anthelmintic therapies.
- The WHO-recommended treatment regimen for clonorchiasis is oral praziquantel 25 mg/kg three times a day for 2 days. Alternative agents include albendazole or tribendimidine.

## Data Availability

Data may be made available on reasonable request from the corresponding author.

## References

[1] Harrington D, Lamberton PHL, McGregor A. 2017. Human liver flukes. Lancet Gastroenterol Hepatol 2:680–689. doi:10.1016/S2468-1253(17)30111-5 28786389

[2] Meyers W, Neafe R, Marty A, Wear D. 2000. Pathology of infectious diseases. 1: helminthiases. Armed Forces Institute Of Pathology, Washington.

[3] Tang Z-L, Huang Y, Yu X-B. 2016. Current status and perspectives of Clonorchis sinensis and clonorchiasis: epidemiology, pathogenesis, omics, prevention and control. Infect Dis Poverty 5:71. doi:10.1186/s40249-016-0166-1 27384714 PMC4933995

[4] Qian M-B, Utzinger J, Keiser J, Zhou X-N. 2016. Clonorchiasis. The Lancet 387:800–810. doi:10.1016/S0140-6736(15)60313-0 26299184

[5] Qian M-B, Li H-M, Jiang Z-H, Yang Y-C, Lu M-F, Wei K, Wei S-L, Chen Y, Zhou C-H, Chen Y-D, Zhou X-N. 2021. Severe hepatobiliary morbidity is associated with Clonorchis sinensis infection: the evidence from a cross-sectional community study. PLoS Negl Trop Dis 15:e0009116. doi:10.1371/journal.pntd.0009116 33507969 PMC7880442

[6] Qian M-B, Chen Y-D, Liang S, Yang G-J, Zhou X-N. 2012. The global epidemiology of clonorchiasis and its relation with cholangiocarcinoma. Infect Dis Poverty 1:4. doi:10.1186/2049-9957-1-4 23849183 PMC3710150

[7] Qian M-B, Chen Y-D, Yan F. 2013. Time to tackle clonorchiasis in China. Infect Dis Poverty 2:4. doi:10.1186/2049-9957-2-4 23849773 PMC3707093

[8] Seah SK. 1973. Intestinal parasites in Chinese immigrants in a Canadian city. J Trop Med Hyg 76:291–293. 4586094

[9] Attwood HD, Chou ST. 1978. The longevity of Clonorchis sinensis. Pathology (Phila) 10:153–156. doi:10.3109/00313027809063494 355989

[10] Kan SP, Cheah JS. 1970. A case of clonorchiasis in Singapore. J Trop Med Hyg 73:143–144. 5463787

[11] O’Rourke A. 2024. A systematic review of the effects of hepatitis B and C virus on the progression of liver fluke infection to liver cancer. Trop Dis Travel Med Vaccines 10:6. doi:10.1186/s40794-023-00215-8 38486298 PMC10941421

[12] Dong H, Liao Y, Shang M, Fu Y, Zhang H, Luo M, Hu B. 2024. Effects of co-infection with Clonorchis sinensis on T cell exhaustion levels in patients with chronic hepatitis B. J Helminthol 98:e13. doi:10.1017/S0022149X23000871 38263743

[13] Li W, Dong H, Huang Y, Chen T, Kong X, Sun H, Yu X, Xu J. 2016. Clonorchis sinensis co-infection could affect the disease state and treatment response of HBV patients. PLoS Negl Trop Dis 10:e0004806. doi:10.1371/journal.pntd.0004806 27348302 PMC4922651

[14] Li Y-K, Zhao J-F, Yang C-L, Zhan G-H, Zhang J, Qin S-D, Zhou M, Li M-J, Huang J-T, Kong F-Y, Huang H, Chen J-H, Xiang B-D. 2023. Effects of Clonorchis sinensis combined with Hepatitis B virus infection on the prognosis of patients with hepatocellular carcinoma following Hepatectomy. PLoS Negl Trop Dis 17:e0011012. doi:10.1371/journal.pntd.0011012 36638133 PMC9879467

[15] Gao Y, Li Y, Liu X, Zhang T, Yu G, Wang Y, Shi Y, Chi X, Wang X, Gao X, et al.. 2020. High prevalence of Clonorchis sinensis infections and coinfection with hepatitis virus in riverside villages in northeast China. Sci Rep 10:11749. doi:10.1038/s41598-020-68684-x 32678224 PMC7366707

[16] Qian M-B, Patel C, Palmeirim MS, Wang X, Schindler C, Utzinger J, Zhou X-N, Keiser J. 2022. Efficacy of drugs against clonorchiasis and opisthorchiasis: a systematic review and network meta-analysis. Lancet Microbe 3:e616–e624. doi:10.1016/S2666-5247(22)00026-X 35697047

[17] Vinh HQ, Phimpraphai W, Tangkawattana S, Smith JF, Kaewkes S, Dung DT, Duong TT, Sripa B. 2017. Risk factors for Clonorchis sinensis infection transmission in humans in northern Vietnam: a descriptive and social network analysis study. Parasitol Int 66:74–82. doi:10.1016/j.parint.2016.11.018 27939296 PMC5292293

